# Supplementary material for: The Structural Effects of Phosphorylation of Protein Arginine Methyltransferase 5 on Its Binding to Histone H4
Source: Int J Mol Sci. 2022 Sep 26;23(19):11316. doi: 10.3390/ijms231911316 (PMC9569665; doi:10.3390/ijms231911316)
Supplement: Supplementary file 1 [file ijms-23-11316-s001.zip › ijms-1904511-supplementary.pdf]

Supplementary Information

The structural effects of phosphorylation of protein arginine methyltransferase 5 on its binding to histone H4

Rita Börzsei[1,2], Bayartsetseg Bayarsaikhan[1], Balázs Zoltán Zsidó[1,2], Beáta Lontay[3], Csaba Hetényi[1,2*]

[1]*Department of Pharmacology and Pharmacotherapy, Medical School, University of Pécs, Hungary.*
[2]*János Szentágothai Research Centre & Centre for Neuroscience, University of Pécs, Hungary.*
[3]*Department of Medical Chemistry, Faculty of Medicine, University of Debrecen, 4032 Debrecen, Hungary.*

Table of Contents

Table S1 Human and non-human PRMT5 complexes collected from PDB Database………2

Table S2 Lenard-Jones and Coulumb type interaction energy of the unmodified and phosphorylated H4-PRMT5-MEP50 complexes in P1 and P2 conformation. ……………….6

Table S3 Interacting H4 residues within 3.5 Å distance from DNA in the structure of nucleosome core particle………………..…………………………………………………7

Figure S1 RMSD plots of histone H4, PRMT5 and MEP50 during MD simulations……….8

Figure S2 RMSD of Helix 3 of histone H4 and its conformations with maximum and minimum RMSD value………………………………………………………………..…9

Figure S3 Changing of the distance between the hydroxyl O atoms of R40, R45 (histone H4) and phosphate P atom of pT80 (PRMT5) during the MD simulation…………10

Figure S4 The representative structure of the unmodified H4-PRMT5-MEP50 complex in the pre-product state……………………………………..…………………10

Figure S5 Representative structure of histone H4 protein in complex with PRMT5P in the pre-product state……………………………………………………….…11

References……………………………………………………………………………...12

Table S1 Human and non-human PRMT5 complexes collected from PDB Database.

PDB code	Release date	Title	Target	Ligand	Method	Reso-lution	Reference
Human PRMT5 complexes							
7U30	2022	PRMT5:MEP50 complexed with cyclonucleoside Compound 1	PRMT5: MEP50	LB3 707 - PRMT5 inhibitor	X-ray diffraction	2.6	[1]
7s1p	2022	PRMT5/MEP50 crystal structure with sinefungin bound	PRMT5: MEP50	MRTx1719 - PRMT5 inhibitor	X-ray diffraction	2.21	[2]
7bo7	2022	Crystal structure of the human PRMT5:MEP50 complex with JNJB44355437	PRMT5: MEP50	PRMT5 inhibitor	X-ray diffraction	2.83	Brown et al. (To be published)
7ses	2022	PRMT5/MEP50 with compound 29 bound	PRMT5: MEP50	PRMT5 inhibitor	X-ray diffraction	2.5	[2]
7ser	2022	PRMT5/MEP50 with compound 30 bound	PRMT5: MEP50	PRMT5 inhibitor	X-ray diffraction	2.14	[2]
7s1s	2022	PRMT5/MEP50 crystal structure with MTA and MRTX-1719 bound	PRMT5: MEP50	PRMT5 inhibitor	X-ray diffraction	2.62	[2]
7s1r	2022	PRMT5/MEP50 crystal structure with MTA and a phthalazinone inhibitor bound (compound (M)-31)	PRMT5: MEP50	PRMT5 inhibitor	X-ray diffraction	2.10	[2]
7s1Q	2022	PRMT5/MEP50 crystal structure with MTA and a phthalazinone inhibitor bound (Compound 9)	PRMT5: MEP50	PRMT5 inhibitor	X-ray diffraction	2.78	[2]
7s0u	2022	PRMT5/MEP50 crystal structure with MTA and	PRMT5: MEP50	PRMT5 inhibitor	X-ray diffraction	2.01	[2]

		phthalazinone fragment bound					
7mxn	2021	PRMT5(M420T mutant):MEP50 complexed with inhibitor PF-06939999	PRMT5 (M420T mutant): MEP	PRMT5 inhibitor	X-ray diffraction	2.55	[3]
7mxg	2021	PRMT5(M420T mutant):MEP50 complexed with inhibitor PF-06855800	PRMT5 (M420T mutant): MEP	PRMT5 inhibitor	X-ray diffraction	2.395	[3]
7mxc	2021	PRMT5:MEP50 complexed with adenosine	PRMT5: MEP50	adenosine	X-ray diffraction	2.41	[3]
7mxa	2021	PRMT5:MEP50 complexed with inhibitor PF-06855800	PRMT5: MEP50	PRMT5 inhibitor	X-ray diffraction	2.713	[3]
7mx7	2021	PRMT5:MEP50 complexed with inhibitor PF-06939999	PRMT5: MEP50	PRMT5 inhibitor	X-ray diffraction	2.49	[3]
7kid	2021	PRMT5:MEP50 complexed with 5,5-bicyclic inhibitor Compound 72	PRMT5: MEP50	PRMT5 inhibitor	X-ray diffraction	2.5	[4]
7kic	2021	PRMT5:MEP50 complexed with 5,5-bicyclic inhibitor Compound 34	PRMT5: MEP50	PRMT5 inhibitor	X-ray diffraction	2.43	[4]
7kib	2021	PRMT5:MEP50 complexed with 5,5-bicyclic inhibitor Compound 4	PRMT5: MEP50	PRMT5 inhibitor	X-ray diffraction	2.52	[4]
7l1g	2021	PRMT5-MEP50 complexed with SAM	PRMT5: MEP50	SAM	X-ray diffraction	2.47	[5]
7m05	2021	CryoEM structure of PRMT5 bound to covalent PBM-	PRMT5: MEP50	PRMT5 inhibitor	Cryo-EM	2.39	[6]

		site inhibitor BRD-6988					
6ugh	2020	Cryo-EM structure of the apo form of human PRMT5:MEP50 complex at a resolution of 3.4 angstrom	PRMT5: MEP50		Cryo-EM	3.4	Zhou (to be published)
6v0p	2020	PRMT5 complex bound to covalent PBM inhibitor BRD6711	PRMT5: MEP50	PRMT5 inhibitor	X-ray diffraction	1.88	[6]
6v0o	2020	PRMT5 bound to the PBM peptide from pICln	PRMT5: MEP50: PBM	sinefungin	X-ray diffraction	2.86	[7]
6v0n	2020	PRMT5 bound to PBM peptide from Riok1	PRMT5: MEP50: Riok1 PBM	sinefungin	X-ray diffraction	2.11	[7]
6uxy	2020	PRMT5:MEP50 complexed with allosteric inhibitor Compound 8	PRMT5: MEP50	PRMT5 inhibitor	X-ray diffraction	2.57	[8]
6uxx	2020	PRMT5:MEP50 complexed with allosteric inhibitor Compound 1a	PRMT5: MEP50	PRMT5 inhibitor	X-ray diffraction	2.69	[8]
6rlq	2020	Crystal structure of the human PRMT5:MEP50 complex with JNJ45031882	PRMT5: MEP50	PRMT5 inhibitor	X-ray diffraction	2.53	Brown et al. (To be published)
6rll	2020	Crystal structure of the human PRMT5:MEP50 complex with JNJ44064146	PRMT5: MEP50	PRMT5 inhibitor	X-ray diffraction	2.22	Brown et al. (To be published)
6k1s	2019	Discovery of potent and selective covalent protein arginine methyltransferase	PRMT5: MEP50	PRMT5 inhibitor	X-ray diffraction	2.6	[9]

		(PRMT5) inhibitors					
6ckc	2018	Structure of PRMT5:MEP50 in complex with LLY-283, a potent and selective inhibitor of PRMT5, with antitumor activity	PRMT5: MEP50	PRMT5 inhibitor	X-ray diffraction	2.8	[10]
5c9z	2016	Crystal structure of PRMT5:MEP50 with EPZ015866 and sinefungin	PRMT5: MEP50	PRMT5 inhibitor	X-ray diffraction	2.36	Boriack-Sjodin (To be published)
5fa5	2016	Crystal structure of PRMT5:MEP50 in complex with MTA and H4 peptide	PRMT5: MEP50	H4, MTA	X-ray diffraction	2.34	[11]
5em m	2016	Crystal structure of PRMT5:MEP50 with Compound 15 and sinefungin	PRMT5: MEP50	PRMT5 inhibitor	X-ray diffraction	2.37	[12]
5eml	2016	Crystal structure of PRMT5:MEP50 with Compound 10 and SAM	PRMT5: MEP50	PRMT5 inhibitor	X-ray diffraction	2.39	[12]
5emk	2016	Crystal structure of PRMT5:MEP50 with Compound 9 and sinefungin	PRMT5: MEP50	PRMT5 inhibitor	X-ray diffraction	2.52	[12]
5emj	2016	Crystal structure of PRMT5:MEP50 with Compound 8 and sinefungin	PRMT5: MEP50	PRMT5 inhibitor	X-ray diffraction	2.273	[12]
4x63	2015	Crystal structure of PRMT5:MEP50 with EPZ015666 and SAH	PRMT5: MEP50	PRMT5 inhibitor	X-ray diffraction	3.05	[13]

4x61	2015	Crystal structure of PRMT5:MEP50 with EPZ015666 and SAM	PRMT5: MEP50	PRMT5 inhibitor	X-ray diffraction	2.85	[13]
4x60	2015	Crystal structure of PRMT5:MEP50 with EPZ015666 and sinefungin	PRMT5: MEP50	PRMT5 inhibitor	X-ray diffraction	2.35	[13]
4gqb	2012	Crystal structure of the human PRMT5:MEP50 Complex	PRMT5: MEP50	H4	X-ray diffraction	2.06	[14]
Non human PRMT complexes							
4g56	2012	Crystal Structure of full length PRMT5/MEP50 complexes from Xenopus laevis	PRMT5: MEP50		X-ray diffraction	2.95	[15]
3ua4	2011	Crystal Structure of Protein Arginine Methyltransferase PRMT5	PRMT5: MEP50		X-ray diffraction	3.00	[16]
3ua3	2011	Crystal Structure of Protein Arginine Methyltransferase PRMT5 in complex with SAH	PRMT5: MEP50	SAH	X-ray diffraction	3.00	[16]

Table S2 Lenard-Jones (LJ) and Coulumb type (Cb) interaction energy of the unmodified and phosphorylated H4-PRMT5-MEP50 complexes in P1 and P2 conformation. P1 and P2 representative structures were selected by cluster analysis (grey backround) and in the case of P2 also on the basis of E_{inter} (white), respectively. See also Methods for details. Both types of representatives of P2 complex structures show lower intermolecular interaction energy than the P1 structures.

**Complex**		**LJ**	**Cb**	**E_{inter}**
H4-PRMT5-MEP50	P1	-148.7	-134.1	**-282.8**
	P2	-201.4	-168.5	**-369.9**
	P2	-191.7	-167.3	**-359.0**

H4-PRMT5P-MEP50	P1	-190.3	-136.7	**-327.0**
	P2	-192.3	-153.7	**-346.0**
	P2	-184.4	-174.8	**-359.2**

Table S3 Interacting H4 residues within 3.5 Å distance from DNA in the structure of nucleosome core particle (PDB code: 1kx5). Amino acids taking place in DNA binding in the case of both histone H4 peptides (chain ID B and F) and determined as DNA binding domain of histone H4 in nucleosome are highlighted with grey.

Histone H4 residues	Residues taking place in DNA binding for	
	Chain B	Chain F
S1		dG+25 I, dG+24 I
G2		dG+25 I
R3		dG+25 I
K5	dT-10 I, dT-9 I	
G6	dT-10 I	
G9	dG+14 J	dA+26 I, dG+27 I
K12		dG+25 I
K16	dG+25 I, dC+15 J, dC+16 J	dG+24 I, dG+25 I
R17	dC+15 J, dC+16 J	dT-22 J
H18	dC+16 J	dC-21 J
R19	dC+16 J, dT+17 J	dT-22 J
T30		dT-12 J
P32	dA-13 I	dA-13 J
R35	dG+8 J	
R36	dA-13 I	dA-13 J
R45	dT+7 J, dG+8 J, dG-3*	dT+7 I, dG+8 I
I46	dT+7J, dG+8 J	dT+7 I, dG+8 I
S47		dT+7 I
G48	dT+7 J	dT+7 I
R78	dC+28 J	
K79	dG+27 J, dC+28 J	dG+27 I, dC+28 I
T80	dC+28 J	dC+28 I

* distance is 4.8 Å

Figure S1 RMSD for Cα atoms of histone H4 (A) compared to the initial conformation of the peptide during the 1000 ns-long MD simulation. RMSD for Cα atoms of PRMT5 (B) and MEP50 (C) after a least squares fitting to the MEP50 structure during the 580 ns-long MD simulation.

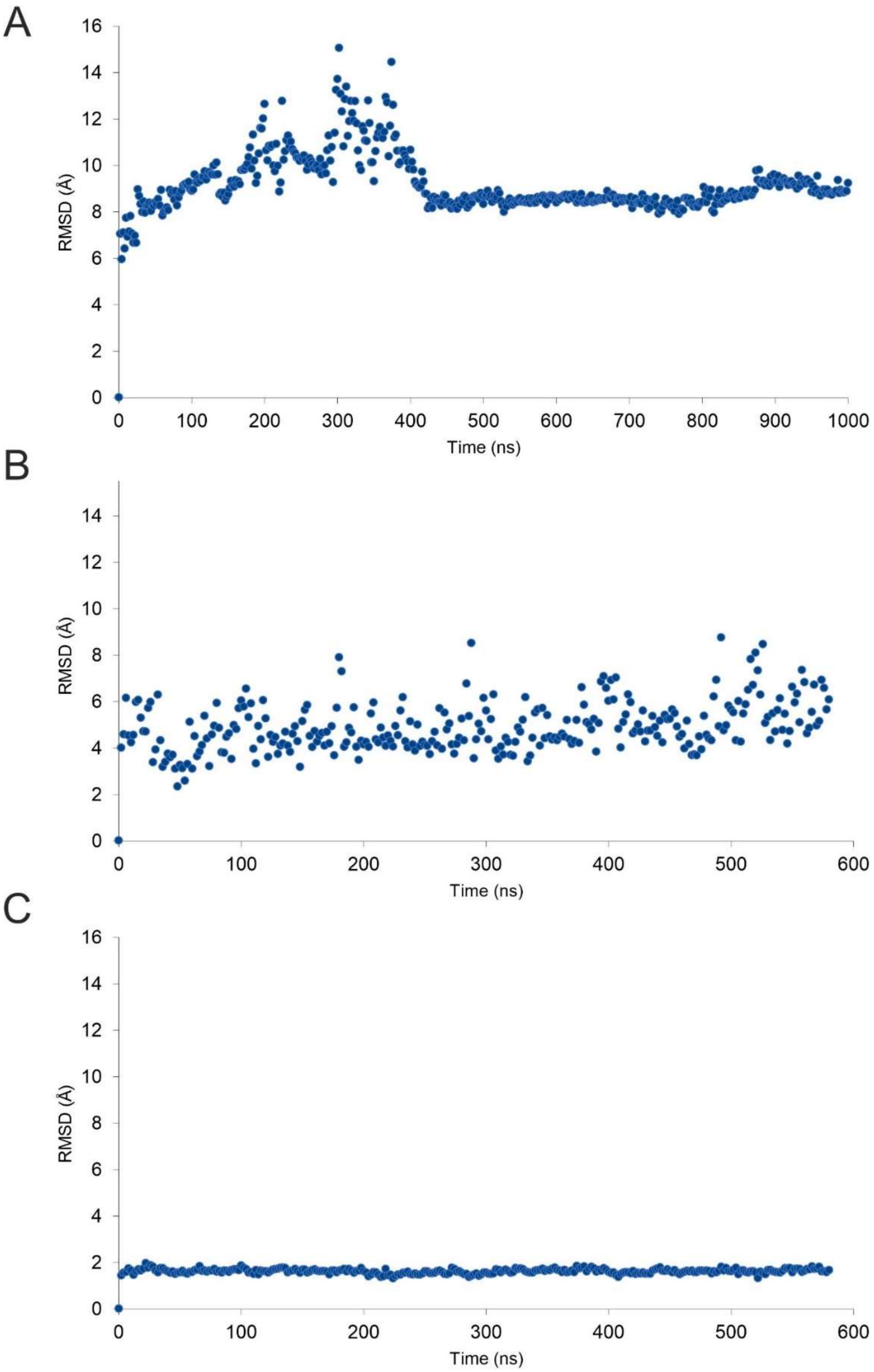

Figure S2 (**A**) Cα RMSD of Helix 3 of histone H4. Time points with the minimum and the maximum RMSD value are highlighted with red. (**B**) Histone H4 structures (grey, cartoon) with the minimum and the maximum RMSD value. Position of the helix fracture at residue G56 is represented as spheres, all atom.

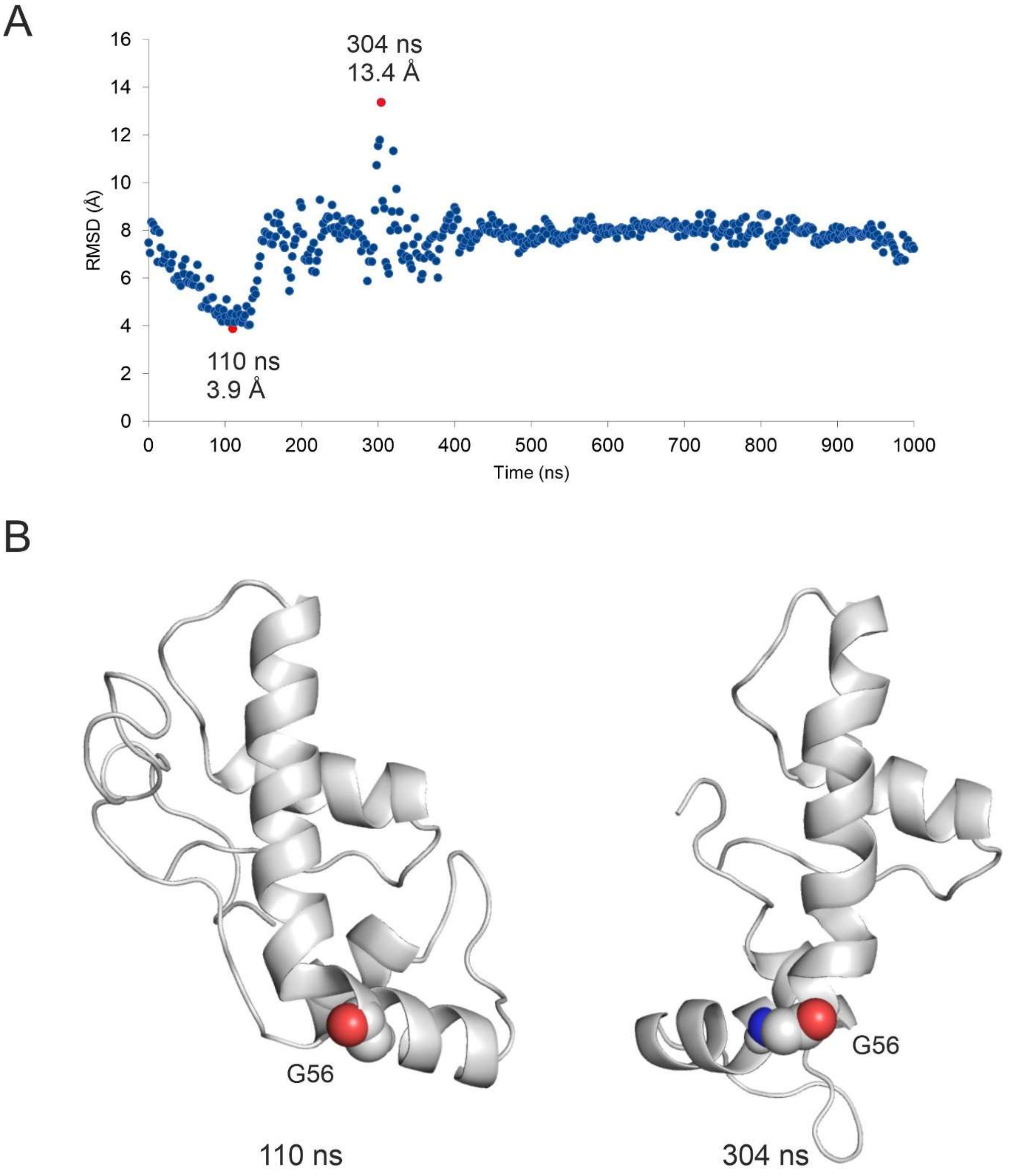

Figure S3 Changing of the distance between the hydroxyl O atoms of R40, R45 (histone H4) and phosphate P atom of pT80 (PRMT5) (d_{O-P}) during the MD simulation.

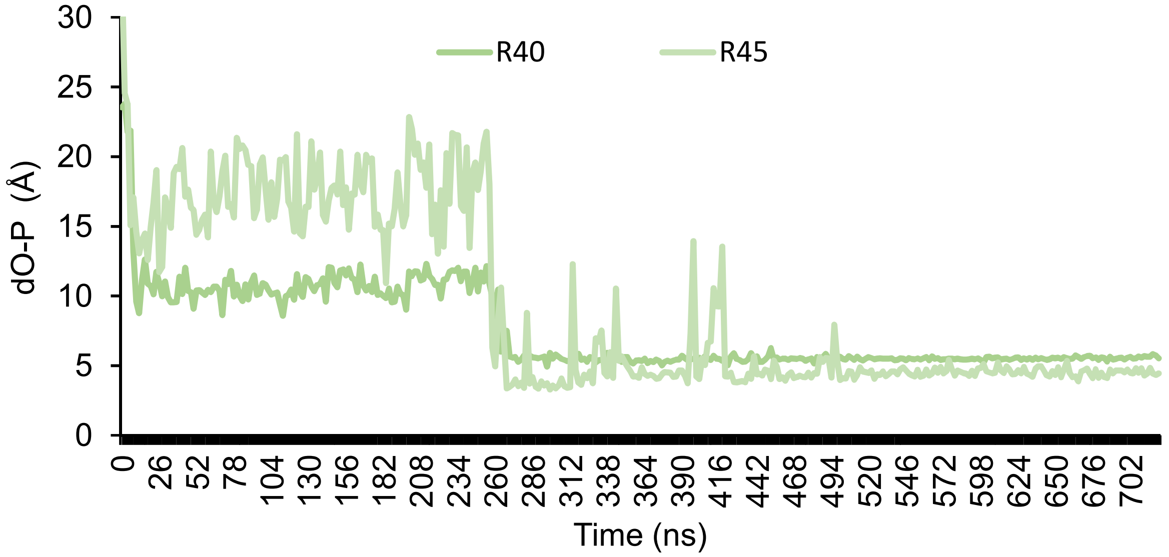

Figure S4 The representative structure of the unmodified H4-PRMT5-MEP50 complex in the pre-product state (P2 in **Fig. 1B**). MEP50 (blue, cartoon) is in direct contact with PRMT5 (grey, cartoon) instead of histone H4 (teal, cartoon).

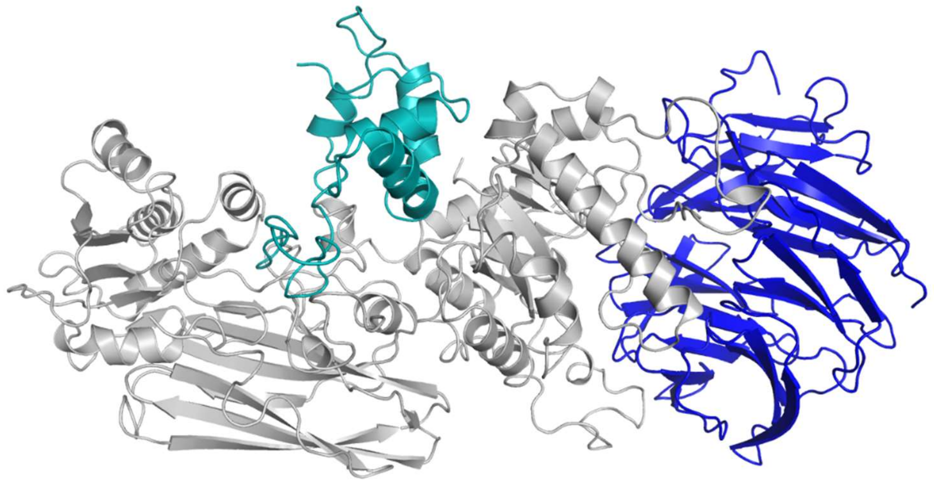

Figure S5 Representative structure of histone H4 protein (grey and teal, cartoon) in complex with PRMT5$_P$ in the pre-product state. Helix 3 (teal) adopts a mostly linear conformation similar to the apo form of H4.

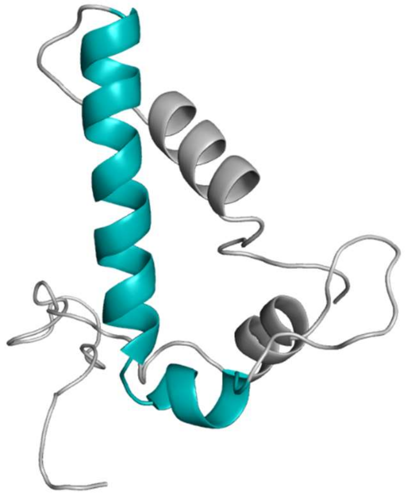

References

1. Kawamura, S.; Palte, R.L.; Kim, H.-Y.; Saurí, J.; Sondey, C.; Mansueto, M.S.; Altman, M.D.; Machacek, M.R. Design and Synthesis of Unprecedented 9- and 10-Membered Cyclonucleosides with PRMT5 Inhibitory Activity. *Bioorg. Med. Chem.* **2022**, *66*, 116820, doi:10.1016/j.bmc.2022.116820.
2. Smith, C.R.; Aranda, R.; Bobinski, T.P.; Briere, D.M.; Burns, A.C.; Christensen, J.G.; Clarine, J.; Engstrom, L.D.; Gunn, R.J.; Ivetac, A.; et al. Fragment-Based Discovery of MRTX1719, a Synthetic Lethal Inhibitor of the PRMT5•MTA Complex for the Treatment of MTAP-Deleted Cancers. *J. Med. Chem.* **2022**, *65*, 1749–1766, doi:10.1021/acs.jmedchem.1c01900.
3. Jensen-Pergakes, K.; Tatlock, J.; Maegley, K.A.; McAlpine, I.J.; McTigue, M.; Xie, T.; Dillon, C.P.; Wang, Y.; Yamazaki, S.; Spiegel, N.; et al. SAM-Competitive PRMT5 Inhibitor PF-06939999 Demonstrates Antitumor Activity in Splicing Dysregulated NSCLC with Decreased Liability of Drug Resistance. *Mol. Cancer Ther.* **2022**, *21*, 3–15, doi:10.1158/1535-7163.MCT-21-0620.
4. Quiroz, R.V.; Reutershan, M.H.; Schneider, S.E.; Sloman, D.; Lacey, B.M.; Swalm, B.M.; Yeung, C.S.; Gibeau, C.; Spellman, D.S.; Rankic, D.A.; et al. The Discovery of Two Novel Classes of 5,5-Bicyclic Nucleoside-Derived PRMT5 Inhibitors for the Treatment of Cancer. *J. Med. Chem.* **2021**, *64*, 3911–3939, doi:10.1021/acs.jmedchem.0c02083.
5. Candito, D.A.; Ye, Y.; Quiroz, R.V.; Reutershan, M.H.; Witter, D.; Gadamsetty, S.B.; Li, H.; Saurí, J.; Schneider, S.E.; Lam, Y.; et al. Development of a Flexible and Robust Synthesis of Tetrahydrofuro[3,4-b]Furan Nucleoside Analogues. *J. Org. Chem.* **2021**, *86*, 5142–5151, doi:10.1021/acs.joc.0c02969.
6. McKinney, D.C.; McMillan, B.J.; Ranaghan, M.J.; Moroco, J.A.; Brousseau, M.; Mullin-Bernstein, Z.; O'Keefe, M.; McCarren, P.; Mesleh, M.F.; Mulvaney, K.M.; et al. Discovery of a First-in-Class Inhibitor of the PRMT5–Substrate Adaptor Interaction. *J. Med. Chem.* **2021**, *64*, 11148–11168, doi:10.1021/acs.jmedchem.1c00507.
7. Mulvaney, K.M.; Blomquist, C.; Acharya, N.; Li, R.; Ranaghan, M.J.; O'Keefe, M.; Rodriguez, D.J.; Young, M.J.; Kesar, D.; Pal, D.; et al. Molecular Basis for Substrate Recruitment to the PRMT5 Methylosome. *Mol. Cell* **2021**, *81*, 3481-3495.e7, doi:10.1016/j.molcel.2021.07.019.
8. Palte, R.L.; Schneider, S.E.; Altman, M.D.; Hayes, R.P.; Kawamura, S.; Lacey, B.M.; Mansueto, M.S.; Reutershan, M.; Siliphaivanh, P.; Sondey, C.; et al. Allosteric Modulation of Protein Arginine Methyltransferase 5 (PRMT5). *ACS Med. Chem. Lett.* **2020**, *11*, 1688–1693, doi:10.1021/acsmedchemlett.9b00525.
9. Lin, H.; Wang, M.; Zhang, Y.W.; Tong, S.; Leal, R.A.; Shetty, R.; Vaddi, K.; Luengo, J.I. Discovery of Potent and Selective Covalent Protein Arginine Methyltransferase 5 (PRMT5) Inhibitors. *ACS Med. Chem. Lett.* **2019**, *10*, 1033–1038, doi:10.1021/acsmedchemlett.9b00074.
10. Bonday, Z.Q.; Cortez, G.S.; Grogan, M.J.; Antonysamy, S.; Weichert, K.; Bocchinfuso, W.P.; Li, F.; Kennedy, S.; Li, B.; Mader, M.M.; et al. LLY-283, a Potent and Selective Inhibitor of Arginine Methyltransferase 5, PRMT5, with Antitumor Activity. *ACS Med. Chem. Lett.* **2018**, *9*, 612–617, doi:10.1021/acsmedchemlett.8b00014.
11. Mavrakis, K.J.; McDonald, E.R.; Schlabach, M.R.; Billy, E.; Hoffman, G.R.; deWeck, A.; Ruddy, D.A.; Venkatesan, K.; Yu, J.; McAllister, G.; et al. Disordered Methionine Metabolism in MTAP/CDKN2A-Deleted Cancers Leads to Dependence on PRMT5. *Science* **2016**, *351*, 1208–1213, doi:10.1126/science.aad5944.
12. Duncan, K.W.; Rioux, N.; Boriack-Sjodin, P.A.; Munchhof, M.J.; Reiter, L.A.; Majer, C.R.; Jin, L.; Johnston, L.D.; Chan-Penebre, E.; Kuplast, K.G.; et al. Structure and

Property Guided Design in the Identification of PRMT5 Tool Compound EPZ015666. *ACS Med. Chem. Lett.* **2016**, *7*, 162–166, doi:10.1021/acsmedchemlett.5b00380.

13. Chan-Penebre, E.; Kuplast, K.G.; Majer, C.R.; Boriack-Sjodin, P.A.; Wigle, T.J.; Johnston, L.D.; Rioux, N.; Munchhof, M.J.; Jin, L.; Jacques, S.L.; et al. A Selective Inhibitor of PRMT5 with in Vivo and in Vitro Potency in MCL Models. *Nat. Chem. Biol.* **2015**, *11*, 432–437, doi:10.1038/nchembio.1810.
14. Antonysamy, S.; Bonday, Z.; Campbell, R.M.; Doyle, B.; Druzina, Z.; Gheyi, T.; Han, B.; Jungheim, L.N.; Qian, Y.; Rauch, C.; et al. Crystal Structure of the Human PRMT5:MEP50 Complex. *Proc. Natl. Acad. Sci. U. S. A.* **2012**, *109*, 17960–17965, doi:10.1073/pnas.1209814109.
15. Ho, M.-C.; Wilczek, C.; Bonanno, J.B.; Xing, L.; Seznec, J.; Matsui, T.; Carter, L.G.; Onikubo, T.; Kumar, P.R.; Chan, M.K.; et al. Structure of the Arginine Methyltransferase PRMT5-MEP50 Reveals a Mechanism for Substrate Specificity. *PLOS ONE* **2013**, *8*, e57008, doi:10.1371/journal.pone.0057008.
16. Sun, L.; Wang, M.; Lv, Z.; Yang, N.; Liu, Y.; Bao, S.; Gong, W.; Xu, R.-M. Structural Insights into Protein Arginine Symmetric Dimethylation by PRMT5. *Proc. Natl. Acad. Sci.* **2011**, *108*, 20538–20543, doi:10.1073/pnas.1106946108.
